# Tobacco use and nicotine dependency in a cross-sectional representative sample of 18,018 individuals in Andaman and Nicobar Islands, India

**DOI:** 10.1186/1471-2458-12-515

**Published:** 2012-07-10

**Authors:** Vivek Benegal, Attayuru Purushottaman Sugunan, Panniyammakal Jeemon, Nagalla Balakrishna, Kandavelu Thennarusu, Dhanasekara Pandian, Kasturi S Pesala

**Affiliations:** 1 Regional Medical Research Centre (ICMR), Port Blair-744101, Andaman & Nicobar Islands, India; 2 Current address: Centre for Chronic Disease Control, Safdarjung Development Area, New Delhi-110016, India; 3 National Institute of Mental Health and Neuro Sciences, Bangalore-560029, Karnataka, India; 4 Institute of Cardiovascular and Medical Sciences, University of Glasgow, Glasgow, UK; 5 Public Health Foundation of India, New Delhi-110016, India; 6 National Institute of Nutrition, Hyderabad-500007, Andhra Pradesh, India; 7 Indira Gandhi National Open University, Port Blair-744101, Andaman & Nicobar Islands, India

## Abstract

**Background:**

Data on prevalence, pattern of tobacco use, proportion of population dependent on nicotine and their determinants are important for developing and implementing tobacco control strategies. The aim of the study was to estimate the prevalence and determinants of tobacco use and nicotine dependency.

**Methods:**

A cross-sectional survey among a representative sample of 18,018 individuals in the age group of >=14 years was conducted in the Union Territory of Andaman and Nicobar Islands during 2007–09. A structured questionnaire, a modified version of an instrument which was used successfully in several multi-country epidemiological studies of the World Health Organisation, was used to survey individual socio-demographic details, known co-morbid conditions, tobacco use and alcohol use. Fagerström Test for Nicotine Dependence (FTND) was used to estimate nicotine dependence.

**Results:**

The response rate of our survey was 97% (18,018/18,554). Females (n = 8,888) were significantly younger than males (34.3 + 14.6 Vs 36.2 + 15.4 years). The prevalence of current tobacco use in any form was 48.9% (95% CI: 48.2–49.6). Tobacco chewing alone was prevalent in 40.9% (95% CI: 40.1–41.6) of the population. While one tenth of males (9.7%, 95% CI: 9.1–10.4) were nicotine dependent, it was only 3% (95% CI: 2.7–3.4) in females. Three fourth of the tobacco users initiated use of tobacco before reaching 21 years of age. Age, current use of alcohol, poor educational status, marital status, social groups, and co-morbidities were the main determinants of tobacco use and nicotine dependence in the population.

**Conclusion:**

The high prevalence of tobacco use especially the chewing form of tobacco in the Union Territory of Andaman and Nicobar Islands and the differences in prevalence and pattern of tobacco use and nicotine dependency observed across subgroups warrants implementation of culturally specific tobacco control activities in this population.

## Background

The burden of tobacco associated epidemic continues to worsen societies and economies as the combined costs of tobacco-related death and related productivity losses, healthcare expenditures, employee absenteeism, and widespread environmental harm are responsible for draining $500 billion from the global economy each year [1]. Although, tobacco smoking rates are decreasing in the industrialized countries over the past two decades, a dramatic increase in use of tobacco products is documented in the member countries of developing world [1].

While tobacco use among men in the age group of 15–54 years increased from 47.4% to 61.8% in India over the period of 1998–2005 in rural areas, it was increased from 33.6% to 50.3% in urban areas during the same period [2]. Although, several nationwide prevalence studies like the National Family Health Survey (NFHS) and Global Adult Tobacco Survey (GATS) report proportion of population using tobacco products in India [3,4], nicotine dependence rates are not well documented. Furthermore both the NFHS and GATS did not cover the Union Territory (UT) of Andaman and Nicobar Islands. This region is also inhabited by distinct social groups including aboriginal tribes. Lack of knowledge on prevalence of tobacco use, type of tobacco use and nicotine dependence is clear barrier to tobacco cessation initiatives [5]. Therefore we explored the prevalence and determinants of tobacco use and nicotine dependency in the Union Territory of Andaman and Nicobar Islands in India.

## Methods

### Study settings and population

Andaman and Nicobar Islands is a union territory, an archipelago of more than 500 islands and islets located at longitude of 92 to 94 degree east and latitude of 6 to 14 degree north in the Bay of Bengal, 1200 km away from main land India with 38 inhabited islands [6]. Over 356000 people live in this area and consist of six aboriginal tribes. A tsunami event devastated these islands in December 2004 and a large number of people lost their near relatives, assets, means of livelihood, and displaced from their homes and their homelands predominantly in southern group of islands (Nicobar group of Islands).

A cross sectional population based survey was undertaken to assess the prevalence, pattern and determinants of tobacco use and nicotine dependency in the populations of Andaman and Nicobar Islands during 2007–09. The study was approved by the Institutional Ethics Committee (IEC) of the Regional Medical Research Centre (RMRC) of the Indian Council of Medical Research (ICMR), Port Blair, Andaman and Nicobar Island. The survey was carried out in the populations of archipelago which fall into one of five prominent social groupings, namely: 1] The Nicobarese tribal people, who constitute the indigenous population of the archipelago; 2] The Ranchi tribes-re-settled tribal populations from Jharkhand region; 3] People from the mainland who settled in the islands before 1942; 4] Later settlers mainly from Bangladesh, rehabilitated by Government of India under various rehabilitation schemes; 5] non-settlers belonging to Tamil, Telugu, and Malayalam native speaking people from the mainland temporarily living in the islands for business and employment.

The study used a multistage cluster random sampling method to survey 18,000 individuals in the age group of 14 years or more. In the first stage 70 of 204 revenue villages were chosen randomly and in the second stage every third household in these selected villages were chosen by systematic random sampling, first household being a random choice. In Car Nicobar Island all 308 ‘Tuhets’ (extended joint families) were enlisted and 50 Tuhets were chosen by simple random choice. All the members aged 14 and above in these selected ‘Tuhets’ or households were interviewed.

### Study measurements

A structured questionnaire was used to survey the family composition, individual socio-demographic details, known co-morbid conditions, tobacco use and alcohol use. The questionnaire used was a modified version of an instrument which was used successfully in several multi-country epidemiological studies of the World Health Organisation, i.e., Gender, Alcohol and Culture: an International Study (GENACIS) [7]. Piloting of the instrument was carried out to make adaptations to the culturally and socially complex environment of Andaman and Nicobar Islands. The following forms of tobacco use were captured using the survey instrument; *Cigarettes, Bidi, Cigar, Gutkha* [a preparation of crushed areca nut (also called betel nut), tobacco, catechu, paraffin, lime and sweet or savory flavorings], *Zardapan* [betel squid consisting of betel leaf, lime, betel nut and processed, flavoured tobacco sold as zarda which is chewed in the mouth for about 5–8 minutes and spit], *Kagazpan* [a mix of areca nut pieces, lime and processed, flavoured tobacco which is chewed similarly like zardapan and spit], *Khaini* [flavored raw tobacco mixed with lime which is kept below the tongue, lips or in touch with oral mucosa for about 5–10 minutes and spit] and *Sookha* [Non-flavored raw tobacco mixed with lime and used like khaini].

General Health Questionnaire (GHQ-12) was used to assess the mental health status of the individual. It is a mental health scale with 12 items which focuses on breaks in normal functioning, rather than lifelong traits and concerns itself with two major classes of phenomenon a] inability to carry out one’s normal healthy functions and b] appearance of new phenomena of a distressing nature. The scale asks whether the respondent has experienced a particular symptom or behaviour recently. Each item is rated on a four-point scale (less than usual, no more than usual, rather more than usual, or much more than usual); and when using the GHQ-12, it gives a total score of 36. GHQ is a good measure of psychological well being in the population [8-10], with relatively high sensitivity and specificity in Indian origin population in UK [11]. Furthermore, the Tamil (regional language in Tamilnadu state of mainland India) version of the 12 item general health questionnaire has shown relatively high sensitivity of 87.4 and a specificity of 79.2 with a cronbach’s alpha of 0.86 and spilt half – reliability of 0.83 among south Indian rural population [12].

Self reported physical and mental morbidities of the respondents during the last one year were also recorded using a structured questionnaire. To screen for presence of Post Traumatic Stress Disorder (PTSD) as a psychological sequel of the devastating Tsunami, a Trauma Screening Questionnaire (TSQ) was used. This self report questionnaire designed for trauma victims in general, consists of 10 items; five are re-experiencing items and five are arousal items taken from the PTSD Symptom Scale. A useful threshold is to ask whether symptoms have occurred at least twice in the past week [13]. When the cut-off is set to require the endorsement of at least six re-experiencing or arousal items in any combination, the overall efficiency of the screening instrument has been found equivalent to that obtained from a comparison of diagnoses yielded by the two most highly regarded interview assessments currently available for PTSD: the Structured Clinical Interview for DSM-IV PTSD module [14] and the Clinician-Administered PTSD Scale [15]. Subjects having a score of 6 and above were considered to be suffering from PTSD.

Fagerström Test for Nicotine Dependence (FTND) questionnaire, a six item questionnaire was used to assess the pattern and severity of tobacco use. Items from both the versions for smoking and smokeless form of tobacco were used in the present study [16,17]. This is a set of 6 questions having a maximum score of 10 which categorises the consumers of tobacco into various grades of dependence [<5 no-dependence; > = 5 dependence]. Although, the DSM/ICD criteria are the gold standard to measure nicotine dependence, they are not widely used in tobacco research as these tools are more time consuming, and need special clinical skills to administer. Therefore these tools have undergone less validation tests than non-DSM/ICD tools in community based interviewer administered questionnaire surveys. One of the widely used non-DSM/ICD criteria for nicotine dependence is Fagerström Nicotine Dependence Test (FNDT) [16,18,19]. FTND assesses the level of dependence of nicotine and the overall score estimates the tobacco liking and may provide a stronger measure of physical dependence.

Alcohol Use Disorders Identification Test (AUDIT), a simple ten-question test developed by the World Health Organization was used to determine alcohol dependence [20]. The test was designed to be used internationally, and was validated in a study using patients from six countries. Questions 1–3 deal with alcohol consumption, 4–6 relate to alcohol dependence and 7–10 consider alcohol related problems. A score of 4 or more out of 12, in questions 4–6 suggests a possibility of alcohol dependence.

The study instruments were reliably translated into Hindi (using the standard translation-back-translation methodology) before administration and pilot tested in the same community to fine tune the questions. The interviews were carried out by 30 trained field workers, under the supervision of one of the investigators. The training sessions were conducted by a team comprised of Psychiatrist, Psychiatric Social Worker, and Epidemiologists. The data collected were cross checked by the investigators by random selection of 30 subjects every week. The collected data were entered into a computer application manually by trained data entry operators. Data cleaning was done once a week.

### Statistical analysis

To compare proportions and group means, the Chi Square test and ‘independent t test’ or one-way analysis of variance (ANOVA) were used, respectively. Bivariate analyses were performed to understand the variables associated with both tobacco use and nicotine dependence and their odds ratios (OR) were calculated.Considering possible interaction between the factors associated with tobacco use or nicotine dependence, multivariate logistic regression models were constructed, retaining all risk factors and interactions that were found to be significantly associated with tobacco use or nicotine dependence in the bivariate analysis. The data analysis was performed using the Statistical Package for Social Studies version 18.0 (SPSS Inc., Chicago, IL, USA).

## Results

### Socio-Demographic characteristics

In total 18,018 individuals (males = 9,130) participated in the survey with a response rate of 97% (18,018/18,554). Females were significantly younger than males (34.3 + 14.6 years Vs 36.2 + 15.4 years). While more than one third (34%) of the population represented the social group ‘settler’, 42% of the study group represented ‘non-settlers’ (Table 1). Nearly one tenth of the study populations were Ranchi tribes and a similar proportion were Nicobarese. The proportion of Pre-42 social group was very small (2.5% each in males and females). Males were more educated than females (p < 0.001) and the mean years of schooling in males (7.71 + 4.6 years) was one year higher than females (6.7 + 5.0 years). The proportion of study population with widowed/divorced/separated status was 6.1% (9.6% in females and 2.7% in males). Nearly two third of males (62.7%) and females (64.7%) were married or living with partner. Significantly higher proportions of males (70%) were regular employees in comparison to females (9%).

**Table 1:** General characteristics of the study population

	**Male (N = 9130)**	**Female (N = 8888)**	P value*
**Age, years (mean, SD)**	36.2 (15.4)	34.3 (14.6)	<0.001**
**Age group, years (n, %)**			
14–19	1225 (13.4)	1286 (14.5)	
20–29	2481 (27.2)	2671 (30.2)	
30–39	1931 (21.2)	2069 (23.4)	
40–49	1557 (17.1)	1369 (15.5)	
50–59	991 (10.9)	777 (8.8)	
60 plus	933 (10.2)	685 (7.7)	<0.001
**Population groups (n, %)**			
Settler	3103 (34.0)	3028 (34.1)	
Non-settler	3904 (42.8)	3607 (40.6)	
Ranchi	1048 (11.5)	980 (11.0)	
Nicobarese	850 (9.3)	1041 (11.7)	
Pre-42	225 (2.5)	232 (2.6)	<0.001
**Education, years of schooling (mean, SD)**	7.71 (4.6)	6.7 (5.0)	<0.001
**Educational status (n, %)**			
0–4 years	1919 (21.0)	2794 (31.4)	
5–10 years	5199 (57.0)	4530 (51.0)	
>10 years	2010 (22.0)	1562 (17.6)	<0.001
**Marital status (n, %)**			
Married/living with partner	5729 (62.8)	5748 (64.7)	
Widowed/Divorced/Seperated	244 (2.7)	856 (9.6)	
Never married	3156 (34.6)	2282 (25.7)	<0.001
**Employement (n, %)**			
Employed	6372 (69.8)	787 (8.9)	
Unemployed	1353 (14.8)	950 (10.7)	
Student	1313 (14.4)	1316 (14.8)	
Home makers	91 (1.0)	5833 (65.6)	<0.001

*Chi Square test.

**Independent ‘t’ test.

### Mental health and clinical characteristics

Poor mental health status was reported in 9.4% of males and 10.6% of females (p = 0.01). Relatively small proportions of males (3.4%) and females (6.6%) were suffering from post traumatic stress disorders (Table 2). Nearly 12% of the study population reported cardiovascular disease, hypertension or diabetes related co-morbidities. Depression, anxiety or suicidal tendencies were reported in 5.3% and 5.9% of males and females, respectively. Nearly 6% of the population was suffering from co-morbidities associated with injuries.

**Table 2:** Self reported co-morbidities, mental health and behavioral characteristics of the study population

	**Men (N = 9130)**	**Women (N = 8888)**	P value*
**Co-morbidities (n, %)**			
CVD/HTN/Diabetes	1079 (11.8)	1067 (12.0)	0.07
Cancer	18 (0.2)	17 (0.2)	1.00
Depression/Anxiety/Suicidal tendency	481 (5.3)	524 (5.9)	0.07
Injuries	495 (5.4)	564 (6.4)	0.009
**Mental Health Status (n, %)**			
Poor mental health status	862 (9.4)	943 (10.6)	0.01
**Post Traumatic Stress Disorder (n, %)**			
PTSD score > 6	313 (3.4)	583 (6.6)	<0.001
**Alcohol use (n, %)**	3168 (34.7)	564 (6.3)	<0.001
**Alcohol dependency (n, %)**	781 (8.6)	79 (0.9)	<0.001
**Tobacco use (n, %)**	5878 (64.4)	2936 (33.0)	<0.001
**Nicotine dependency (n, %)**	889 (9.7)	267 (3.0)	<0.001

*Chi Square test.

### Tobacco use and nicotine dependence

**Figure 1:**
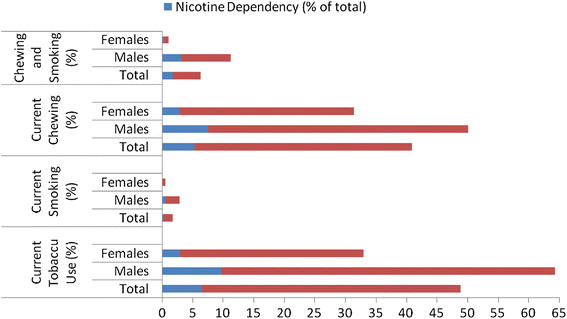
Prevalence of tobacco use and nicotine dependency in the study population.

Nearly one half of the study population (48.9%, 95% CI: 48.2–49.6) was current tobacco users. While one tenth of males (9.7%, 95% CI: 9.1–10.4) were nicotine dependent, it was only 3% (95% CI: 2.7–3.4) in females (<0.001). Tobacco chewing alone was prevalent in 40.9% (95% CI: 40.1–41.6) of the total population. While tobacco in smoking form was prevalent in only 1.7% (95% CI: 1.5–1.9) of the population, combined use of smoking and chewing tobacco was prevalent in 6.3% (95% CI: 6.2–6.4) of the population (Figure 1). Tobacco smoking was in the form of Cigarettes (2.6%, 95% CI: 2.4–2.8), Bidi (5.1%, 95% CI: 4.8–5.4) and Cigar (0.3%, 95% CI: 0.2–0.4). The following forms of tobacco chewing was prevalent in the study population; Gutkha (0.6%, 95% CI: 0.5–0.7), Zardapan (22.6%, 95% CI: 22.0–23.2), Kagazpan (22.1%, 95% CI: 21.5–22.7), Khaini (1.1%, 95% CI: 0.96–1.3) and Sookha (9.0%, 95% CI: 8.6–9.4). While 13% (95% CI: 12.2–13.7) of the tobacco chewers were nicotine dependent, it was 25.5% (23.3–27.8) in tobacco smokers (P < 0.001).

Current tobacco use was significantly higher (p < 0.001) in males (64.4%, 95% CI: 63.4–65.4) in comparison to females (33.3%, 95% CI: 32.1–34.0). It was lowest in the age group of 14–19 years (13.7%, 95% CI: 12.4–15.1) and highest in the 50 plus age group (69.4%, 95% CI: 67.9–70.9). While the prevalence of tobacco use among Nicobarese social group was 83.9% (95% CI: 82.1–85.5), it was lowest in the Pre-42 social group (36.5%, 95% CI: 32.2–41.1). The prevalence rates of tobacco use were 48.1% (95% CI: 46.9–49.4), 40.2% (95% CI: 39.1–41.3) and 53.3% (95% CI: 51.1–55.4) in Settler’s, Non-Settler’s and Ranchi tribe groups, respectively. The prevalence rate was highest in the lowest educational status group (64.3%, 95% CI: 62.9–65.6) and lowest in the highest educational status group (41.4%, 95% CI: 29.8–32.9). Marital status influenced tobacco use as the prevalence rate was lowest (29.9%, 95% CI: 28.7–31.1) in the never married group. While 11.5% (95% CI: 10.3–12.8) of the students were current tobacco users, the prevalence rates were 40.1% (95% CI: 38.8–41.3), 50.2% (95% CI: 48.2–52.3) and 69.5% (95% CI: 68.4–70.5) among home makers, unemployed and employed individuals, respectively. Individual with poor mental health status reported significantly higher tobacco use (55%, 95% CI: 52.7–57.3) in comparison to individuals with normal or good mental health status (48%, 95% CI: 47.2–48.8). Most of the alcohol users were tobacco users (92.6%, 95% CI: 91.7–93.4)) while the prevalence of tobacco use was only 37.4% (36.7–38.2) in non users.

**Figure 2:**
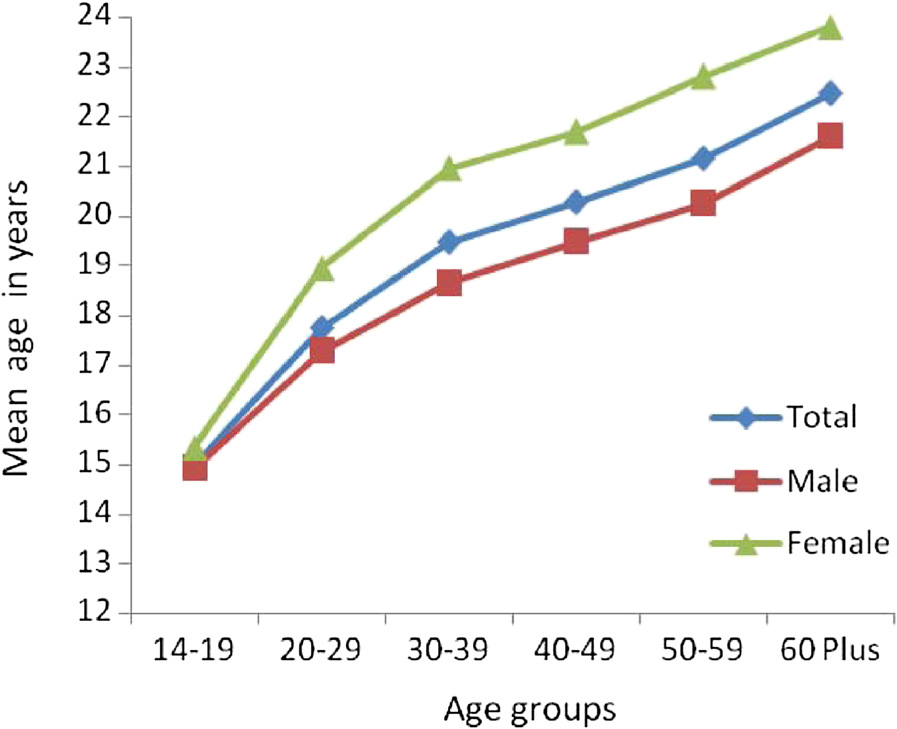
Mean age of initiation of chewing across age groups.

Mean age of initiation of tobacco use was significantly lower in males (19.00 + 5.9 years) in comparison to females (21.34 + 6.8 years). While mean age at initiation of tobacco chewing was 19.65 + 6.1 years, it was 20.44 + 7.8 years for tobacco smoking. The mean age at initiation of tobacco chewing increased with age in both males and females (Figure 2). Three fourth of the tobacco users initiated use of tobacco before reaching 21 years of age.

### Determinants of tobacco use

In the bivariate analysis, female gender (OR = 0.27, 95% CI = 0.26–0.29) in comparison to male gender, Non-settlers (OR-0.72, 95% CI = 0.67–0.77) and Pre-42 group (OR = 0.62, 95% CI = 0.51–0.75) in comparison to Settlers, and unemployed (OR = 0.45, 95% CI = 0.40–0.49), student (OR = 0.06, 95% CI = 0.05–0.07), and home makers (OR = 0.30, 95% CI = 0.27–0.32) in comparison to employed individuals had significantly lower probability to use tobacco products (Table 3). On the other hand the bivariate OR were significantly elevated in 20 plus age group in comparison to individuals in the age group of 14–19 years, Ranchi tribes (OR = 1.23, 95% CI = 1.11–1.36) and Nicobarese (OR = 5.65, 95% CI = 4.95–6.45) in comparison to settlers, middle level education (OR = 2.01, 95% CI = 1.85–2.18) and low level education (OR = 3.92, 95% CI = 3.58–4.30) in comparison to high level education group, widowed/divorced/seperated in comparison to never married individuals (OR = 3.44, 95% CI = 3.01–3.93), individuals with poor mental health status (OR = 1.29, 95% CI = 1.16–1.42) in comparison to individuals with normal or good mental health status, alcohol users (OR = 21.22, 95% CI = 18.67–24.12) in comparison to non-users, and individuals with any co-morbidities (OR = 2.51, 95% CI = 2.32–2.72) in comparison to individuals with no co morbidities.

**Table 3:** Variables associated with tobacco use

	**Tobacco use**	Uni-variate OR (95% CI)*	Multi-variate OR (95% CI)*
**Gender (n,%)**			
Male	5878 (64.4)	1	1
Female	2936 (33.0)	**0.27 (0.26–0.29)**	**0.25 (0.22–0.29)**
**Age group, years (n,%)**			
14–19	345 (13.7)	1	1
20–29	1988 (38.6)	**3.95 (3.50–4.50)**	**2.02 (1.67–2.44)**
30–39	2244 (56.1)	**8.02 (7.00–9.10)**	**3.12 (2.52–3.86)**
40–49	1886 (64.5)	**11.40 (9.90–13.10)**	**3.61 (2.89–4.51)**
50–59	1226 (69.3)	**14.20 (12.20–16.50)**	**3.70 (2.91–4.69)**
60 plus	1124 (69.5)	**14.30 (12.20–16.70)**	**3.64 (2.84–4.68)**
**Population groups (n,%)**			
Settler	2957 (48.2)	1	1
Non-settler	3017 (40.2)	**0.72 (0.67–0.77)**	**0.54 (0.50–0.59)**
Ranchi	1084 (53.5)	**1.23 (1.11–1.36)**	**0.78 (0.68–0.89)**
Nicobarese	1589 (84.0)	**5.65 (4.95–6.45)**	**5.63 (4.78–6.66)**
Pre-42	167 (36.5)	**0.62 (0.51–0.75)**	**0.57 (0.45–0.72)**
**Educational status (n,%)**			
0–4 years	3029 (64.3)	**3.92 (3.58–4.30)**	**2.73 (2.39–3.11)**
5–10 years	4661 (47.9)	**2.01 (1.85–2.18)**	**1.75 (1.56–1.94)**
>10 years	1123 (41.4)	1	1
**Marital status (n,%)**			
Never married	1631 (30.0)	1	1
Married/living with partner	6527 (56.9)	**3.08 (2.87–3.30)**	1.05 (0.92–1.20)
Widowed/Divorced/Seperated	655 (59.5)	**3.44 (3.01–3.93)**	**1.32 (1.08–1.63)**
**Employement (n,%)**			
Employed	4973 (69.5)	1	1
Unemployed	1158 (50.3)	**0.45 (0.40–0.49)**	**0.70 (0.61–0.80)**
Student	303 (11.5)	**0.06 (0.05–0.07)**	**0.32 (0.26–0.39)**
Home makers	2379 (40.2)	**0.30 (0.27–0.32)**	0.96 (0.84–1.01)
**Mental health status (n,%)**			
Normal	7830 (48.3)	1	1
Poor	984 (54.5)	**1.29 (1.16–1.42)**	0.89 (0.79–1.01)
**Post Traumatic Stress Disorder (n,%)**			
PTSD score > 6	465 (51.9)	1	
PTSD score < 6	8349 (48.8)	1.13 (0.99–1.30)	
**Alcohol use (n,%)**			
No	5354 (37.5)	1	1
Yes	3460 (92.7)	**21.22 (18.67–24.12)**	**7.79 (6.78–8.96)**
**Co-morbidities (n, %)**			
CVD/HTN/Diabetes: No	7400 (46.6)	1	
CVD/HTN/Diabetes: Yes	1414 (65.9)	**2.21 (2.01–2.43)**	
Cancer: No	8792 (48.9)	1	
Cancer: Yes	22 (62.9)	1.77 (0.89–3.51)	
Depression/Anxiety/Suicidal tendency: No	8168 (48.0)	1	
Depression/Anxiety/Suicidal tendency: Yes	646 (64.3)	**1.95 (1.71–2.23)**	
Injuries: No	7999 (47.4)	1	
Injuries: Yes	792 (74.8)	**3.30 (2.86–3.80)**	
Any co-morbidities: No	6524 (44.8)	1	1
Any co-morbidities: Yes	2272 (67.0)	**2.51 (2.32–2.72)**	**1.25 (1.13–1.38)**

*Multiple logistic regression results.

In the multi-variate analysis the adjusted OR were significantly lower in females (OR = 0.25, 95% CI = 0.22–0.29) in comparison to males, Non-settlers (OR = 0.54, 95% CI = 0.50–0.59), Ranchi tribes (OR = 0.78, 95% CI = 0.68–0.89), and Pre-42 social group (OR = 0.57, 95% CI = 0.45–0.72) in comparison to settlers, and unemployed (OR = 0.70, 95% CI = 0.61–0.80) and students (OR = 0.32, 95% CI = 0.26–0.39) in comparison to employed individuals (Table 3). The adjusted OR increased linearly with age from 2.0 (95% CI = 1.67–2.44) in the age group 20–29 to 3.7 (95% CI = 2.90–4.70) among individuals with 50 plus age, in comparison to individuals in the 14–19 years age group. Nicobarese had significantly elevated OR (OR = 5.63, 95% CI = 4.78–6.66) after adjustment for all other variables related to tobacco use in comparison to Settlers. Education and tobacco use showed significant inverse relationship with significantly elevated OR in the low education group (OR = 2.73, 95% CI = 2.39–3.11) and middle level education group (OR = 1.75, 95% CI = 1.56–1.94) in comparison to the high education group. Tobacco use was significantly higher in widowed/divorced/seperated (OR = 1.32, 95% CI = 1.08–1.63) in comparison to never married group. Current use of alcohol was associated with current use of tobacco (OR = 7.79, 95% CI = 6.78–8.96). Similarly, presence of any co-morbidities was also associated with current use of tobacco (OR = 1.25, 95% CI = 1.13–1.38).

### Determinants of nicotine dependence

In the multi-variate analysis 30 plus age group (OR = 1.70, 95% CI = 1.34–2.21), social groups [Ranchi tribes (OR = 1.21, 95% CI = 1.00–1.47) and pre-42 social group (OR = 0.47, 95% CI = 0.28–0.80) in comparison to Settlers], low educational status [low (OR = 1.77, 95% CI = 1.40–2.23) and middle educational group (OR = 1.32, 95% CI = 1.07–1.63) in comparison to high educational status], marital status [widowed/separated/divorced (OR = 1.58, 95% CI = 1.12–2.24) in comparison to never married individuals], employment status [unemployed (OR-0.70, 95% CI = 0.56–0.87) and students (OR = 0.13, 95% CI = 0.07–0.24) in comparison to employed individuals], alcohol dependency [alcohol dependent (OR = 4.30, 95% CI = 3.61–5.12) in comparison to alcohol non-dependent] and presence of co morbidities (OR = 1.57, 95% CI = 1.36–1.82) were significantly associated with nicotine dependence (Table 4).

**Table 4:** Variables associated with nicotine dependency among tobacco users

	**Nicotine dependent**	Univariate OR (95% CI)*	Multivariate OR (95% CI)*
**Gender (n,%)**			
Male	889 (9.7)	1	1
Female	267 (3.0)	**0.29 (0.25–0.33)**	0.44 (0.34–0.57)
**Age group, years (n, %)**			
14–29	190 (2.5)	1	1
30–39	274 (6.9)	**2.89 (2.40–3.50)**	**1.61 (1.29–2.02)**
40–49	271 (9.3)	**4.02 (3.32–4.86)**	**1.77 (1.39–2.25)**
50–59	227 (12.8)	**5.79 (4.74–7.08)**	**2.22 (1.72–2.87)**
60 plus	194 (12.0)	**5.36 (4.35–6.60)**	**1.92 (1.44–2.56)**
**Population groups (n, %)**			
Settler	415 (6.8)	1	1
Non-settler	350 (4.7)	**0.67 (0.58–0.78)**	0.64 (0.55–0.74)
Ranchi	232 (11.4)	**1.78 (1.50–2.11)**	**1.21 (1.00–1.47)**
Nicobarese	143 (7.6)	1.13 (0.93–1.37)	0.88 (0.71–1.09)
Pre-42	16 (3.5)	**0.50 (0.30–0.83)**	**0.47 (0.28–0.80)**
**Educational status (n, %)**			
0–4 years	459 (9.7)	**3.08 (2.51–3.78)**	**1.77 (1.40–2.23)**
5–10 years	576 (5.9)	**1.80 (1.47–2.19)**	**1.32 (1.07–1.63)**
>10 years	121 (3.4)	1	1
**Marital status (n, %)**			
Never married	134 (2.5)	1	1
Married/living with partner	920 (8.0)	**3.45 (2.87–4.15)**	1.25 (0.98–1.60)
Widowed/Divorced/Seperated	102 (9.3)	**4.05 (3.10–5.28)**	**1.58 (1.12–2.24)**
**Employement (n, %)**			
Employed	789 (11.0)	1	1
Unemployed	151 (6.6)	**0.57 (0.47–0.68)**	**0.70 (0.56–0.87)**
Student	10 (0.4)	**0.03 (0.02–0.06)**	**0.13 (0.07–0.24)**
Home makers	206 (3.5)	**0.29 (0.25–0.34)**	0.64 (0.48–1.44)
**Mental health status (n, %)**			
Normal	984 (6.1)	1	1
Poor	172 (9.5)	**1.26 (1.01–1.58)**	1.19 (0.98–1.44)
**Post Traumatic Stress Disorder (n, %)**			
PTSD score > 6	54 (6.0)	1	
PTSD score < 6	1102 (6.4)	1.07 (0.81–1.42)	
**Alcohol dependency (n, %)**			
No	867 (5.1)	1	1
Yes	289 (33.6)	**9.51 (8.13–11.13)**	**4.30 (3.61–5.12)**
**Co-morbidities (n, %)**			
No	738 (5.1)	1	1
Yes	417 (12.3)	**2.63 (2.32–2.99)**	**1.57 (1.36–1.82)**

*Multiple logistic regression results.

## Discussion

We studied tobacco use and nicotine dependency in a representative sample of 18,018 individuals in the 14 plus age group in Andaman and Nicobar Islands in India. This is the first such study conducted in the Union Territory of Andaman and Nicobar Islands. The proportion of individuals in our sample population representing the various social groups are similar to the total population proportion of these groups in the Andaman and Nicobar Islands [6]. The sample estimates are therefore very close to the true population estimates. Our study highlights relatively high prevalence of tobacco use in this population (almost 50%). Furthermore, tobacco chewing was the main form of tobacco use.

Almost 58% of the males and 11% of the females were current tobacco users in the National Family Health Survey-3 [NFHS-3] (2005–2006) conducted among individuals aged 15–54 years in 29/35 states and Union Territories in India [3]. In NFHS-3, around 36% of the males and 8% of the females reported use of smokeless tobacco in the form of chewing [3,21]. While 47.9% of the males and 20.3% of the females were current tobacco users in the GATS, the overall prevalence was 34.6%. Furthermore, more than a quarter (26%) of the GATS respondents from India reported use of smokeless tobacco [4]. However, NFHS-3 and GATS did not cover the Union Territory of Andaman and Nicobar Islands [3,4]. The use of tobacco among women in our study is almost four times (33%) higher than that of NFHS-3 survey. Furthermore, in Andaman and Nicobar Islands chewing is the main form of tobacco use and there are different patterns of tobacco chewing prevalent i.e., use of *Zarda Pan, Kagaz pan, Sookha, Khaini*, and *Gutkha*.

Although the overall nicotine dependence rate was only 6.4% in the study population (13.1% among current tobacco users), it was nearly 30% among users of tobacco in mixed form (both chewing and smoking). To the best of our knowledge this is the largest ever community based survey conducted in India to assess the prevalence of nicotine dependence using FTND tool. The FTND is a screening instrument for physical nicotine dependence and is extensively used in various countries. Although, the reliability of this screening tool is questioned in several small studies, it was found to be reliable in different settings and populations [22]. However, further studies of the FTND are needed in the Indian population to assess the validity and reliability of this instrument.

The mean age of initiation of tobacco use was lower in males and in the younger age groups in the study population. The trend observed in males and females with age indicates that the age of onset of tobacco use is coming down in individuals in the newer generation. Furthermore, three fourth of the tobacco users initiated use of tobacco before reaching 21 years of age. This trend is disturbing as it is important to increase the tobacco free years of life in order to reduce the harmful effect of tobacco at the population level.

There were distinct social patterns observed for tobacco use and nicotine dependency in our study. While the prevalence of tobacco use was higher in the Nicobarese tribe, the risk of nicotine dependence was highest among Ranchi groups. Car Nicobar Island in the southern district of Nicobar is totally inhabited by Nicobarese aboriginal tribe. Although, they are aboriginal people, they are no more considered as primitive. More importantly, the overall literacy of Car Nicobar Island is around 75%. Though the tsunami of 2004 devastated the life of tribal living in this island, still they maintain their traditional cultural and social rituals in their daily life [23]. On the other hand, there are over sixty five thousand ‘Ranchi tribes’ (Ranchis) people live in Andaman and Nicobar Islands. During the British rule, since 1918, people from the Chhota Nagpur tribal belt of mainland India were brought to Andaman and they were forced to work as forest labourers. They are known as ‘Ranchis’. Even after India’s independence Ranchis were brought to Andaman and Nicobar Islands as labourers to clear forest areas for settlements. While these communities are recognised as Scheduled Tribes (ST) in their region of origin, they are seen simply as a homogenous group of migrants in Andaman and Nicobar Islands. The Ranchis own no land and rely on irregular labour jobs for survival. While, the STs have fixed quota of benefits for education, employment and other social security measures that are guaranteed under the Indian constitution, the Ranchis are deprived of all these facilities because of their social position. With labour work increasingly insecure, health and education of the community also suffers [24]. While Nicobarese reported to have high prevalence of tobacco use, the risk of nicotine dependence was highest in Ranchis in our study. The phenomenon of ‘anomie’ (the breakdown of social bonds between individuals and loosening of their community ties with fragmentation of social identity and rejection of self regulated values) which is evident in the uprooted "Ranchi tribes" and the subsequent substance abuse may partially explain this paradox [25,26].

Alcohol use was the most significant determinant of tobacco use and nicotine dependence in the study population. Alcohol consumption and tobacco use are closely linked behaviours and importantly people who drink larger amounts of alcohol tend to smoke more cigarettes. Furthermore, smokers who are dependent on nicotine have a 2.7 times greater risk of becoming alcohol dependent than nonsmokers [27]. Therefore, the issue of tobacco control cannot be seen in isolation from control of alcohol abuse.

There was a significant inverse and graded relationship between educational status and tobacco use or nicotine dependence rate. Similar trends in tobacco use are also observed in various population based studies from India [28-30]. Post traumatic stress disorders neither increase the tobacco use nor have an impact on nicotine dependence rate. This data is of particularly important in the context of the devastating Tsunami event happened three years before the survey in the Andaman and Nicobar Islands. PTSD was assessed using trauma screening questionnaire and in general the overall efficiency of this instrument is found to be equivalent to that obtained from DSM-IV PTSD module [13].

The strengths of our study include population based survey methodology covering more than 18,000 individuals, including both males and females in the survey and an overall response rate of 97% (18,018/18,554). The field interviewers visited the houses and Tuhets on multiple occasions to achieve this high response rate. This is probably the highest response rate in a survey of this magnitude conducted in India. While our findings are generlizable to the population of Andaman and Nicobar Islands, the use of standard survey instruments increases the validity of our findings.

### Limitations

The cross-sectional nature of our study and self reported rates of tobacco use are the major limitations of our study. Thus the results demonstrate associations but do not provide evidence for causality. The possibility of underreporting of some of these additive behaviours may be present due to its social unacceptability in certain segments of the society.

### Policy implications

Population data of prevalence estimates of tobacco use, pattern of tobacco use, and determinants of tobacco use and nicotine dependence are important baseline information that influences policy decisions on development and implementation of tobacco control strategies. The high prevalence of tobacco use especially the chewing form of tobacco in the Union Territory of Andaman and Nicobar Islands and the distinct social pattern observed for tobacco use and nicotine dependency warrants implementation of culturally specific tobacco control activities in this population. The relatively higher proportion of females using tobacco products in this region and high prevalence in individuals in the low educational status group also requires special attention while developing tobacco control strategies. Furthermore, special tobacco cessation clinics may be required for individuals who report nicotine dependence. Although, the nicotine dependence prevalence rate is relatively low (6.4%), the absolute number of individuals with nicotine dependency in the population is very high. For example, with the current prevalence estimate more than 20,000 individuals are nicotine dependent in the Union Territory of Andaman and Nicobar Islands. Treatment outcomes for patients addicted to both alcohol and nicotine are generally worse than for people addicted to only one drug, and many treatment providers do not promote smoking cessation during alcoholism treatment. Hence, tobacco control activities should go hand-in-hand with control of alcohol use in this population as the combined use of tobacco and alcohol is very high in this population.

## Conclusion

In a representative sample of 18,018 individuals from the Union Territory of Andaman and Nicobar Islands, the prevalence of current tobacco use in any form was 48.9% (95% CI: 48.2–49.6). Tobacco chewing alone was prevalent in 40.9% (95% CI: 40.1–41.6) of the total population. While tobacco in smoking form was prevalent in only 1.7% (95% CI: 1.5–1.9) of the population, combined use of smoking and chewing tobacco was prevalent in 6.3% (95% CI: 6.2–6.4) of the population. While one tenth of males (9.7%, 95% CI: 9.1–10.4) were nicotine dependent, it was only 3% (95% CI: 2.7–3.4) in females (<0.001). Three fourth of the tobacco users initiated use of tobacco before reaching 21 years of age. Age, current use of alcohol, poor educational status, marital status, social groups, and co-morbidities were the main determinants of tobacco use and nicotine dependence in the population.

## Competing interests

SPM is presently recipient of grant number 1 D43 HD065249 from the Fogarty International Center and the Eunice Kennedy Shriver National Institute of Child Health & Human Development at the National Institutes of Health. PJ is supported by a Wellcome Trust Capacity Strengthening Strategic Award to the Public Health Foundation of India and a Consortium of UK Universities. The authors declare that there are no other competing interests, neither financial nor non financial.

## Authors’ contributions

SPM and APS conceived the study. SPM, VB, APS, and DP designed and executed the study. SPM, VB, APS, PJ, NB, KT, and KSP analysed and interpreted the data. SPM and PJ drafted the manuscript with contributions from all co-authors. All authors read and approved the final version of the manuscript.

## Pre-publication history

The pre-publication history for this paper can be accessed here:

http://www.biomedcentral.com/1471-2458/12/515/prepub
